# The American Medical Library and Intelligencer: A Concentrated Record of Medical Science and Literature

**Published:** 1837-10

**Authors:** 


Art. V.—*The American Medical Library and Intelligencer: a concentrated Record of Medical Science and Literature.* Edited by R. Dunglison, m.d., Professor of the Institutes of Medicine, &c. in Jefferson College. *In semi-monthly Parts.* No. I. to VIII.—8vo. pp. 128 each. *Philadelphia,* 1837.

We have been so long accustomed to the republication, in Brussels, of the French Journals and other medical works from the Parisian press, in a condensed form and at a low price, that we are less surprised at the adoption of a similar practice, in regard to English medical works, by the American publishers. Although such a practice, whereby the interests of the authors of the works thus republished are liable to be greatly compromised, cannot but be regretted, we know not that the individuals who thus republish them, for the benefit of their countrymen and their own, are in any way to be blamed, so long as the present law of national copyright remains unchanged. The temptation to do so is certainly extreme, and the advantages to the readers of the republished works very great. In one of the Numbers of the work now before us, containing a reprint of Sir Benjamin Brodie's Lectures on Local Nervous Affections, we are told that, while the original volume costs, in Philadelphia, one dollar and seventy-five cents, or 175 cents, the same work, as republished, costs only twelve and a half cents. "When this surprising economy is considered, (continues our authority,) with the additional advantage that our *most distant* subscribers can have the best books that are published abroad in a few days after their receipt in the Atlantic cities, the overwhelming value of such a publication as this must be strikingly apparent." And truly so it is to the American reader, although it would probably be more agreeable to English authors to know that, instead of a thousand copies of their works being distributed in their new form, two or three hundred were sold in their original shape and price.

The plan of the work before us is different from any hitherto proposed, yet coming nearer that of the Brussels *Encyclographie* than any other. Instead of republishing the Journals verbatim, like the Encyclographie, the *Library* merely devotes one sheet, or an eighth part of the whole, to the purposes of a Journal, the remainder of its pages being entirely appropriated to the reprint of distinct works or memoirs of a superior class. In the Numbers now before us, the works reprinted are, Wardrop on Bloodletting; Babington on the Blood; Stokes's Lectures on the Practice of Medicine; Brodie on Local Nervous Affections; Itard on Deafness, (translated;) Bright on Renal Disease; Hamilton's Observations in Midwifery; Bricheteau's Medical Clinics; Colles on the Venereal Disease. All these reprints are paged separately, and, as the paper and print are remarkably good, they will truly form, when bound up separately, a most valuable, and comprehensive library for the American practitioner. The price of the whole work is ten dollars per annum.

Notwithstanding some doubts entertained by us, as foreigners, respecting the justice of the international law which permits so serious an invasion of the rights of authors, we cannot but admit that the present publication is calculated to be of immense benefit to our brethren in America; and we shall certainly act more in accordance with the spirit of our beneficent profession in wishing it every success, as a means of promoting the essential good of mankind, than in regarding it as a commercial speculation injurious to the pecuniary interests of individuals. The manner in which the work is arranged and conducted is in the highest degree creditable to its very learned and industrious editor.

